# Supplementary material for: Analysis of Vibrio cholerae genomes identifies new type VI secretion system gene clusters
Source: Genome Biol. 2019 Aug 12;20:163. doi: 10.1186/s13059-019-1765-5 (PMC6691524; doi:10.1186/s13059-019-1765-5)
Supplement: Supplementary file 1 — Figure S1. Depiction of T6SS gene clusters in the BGT49 genome. Figure S2. TleV1 is predicted to share structural homology to the P. aeruginosa Tle1 T6SS lipase. Figure S3. BGT49 genome region containing the Aux 5a cluster. Figure S4. Expression of the tliV1b gene does not neutralize the toxicity of Tat-TleV1. Figure S5. Graphical description of the annotation workflow used in this study and, in part, by the T6SS Predictor. (PDF 1330 kb) [file 13059_2019_1765_MOESM1_ESM.pdf]

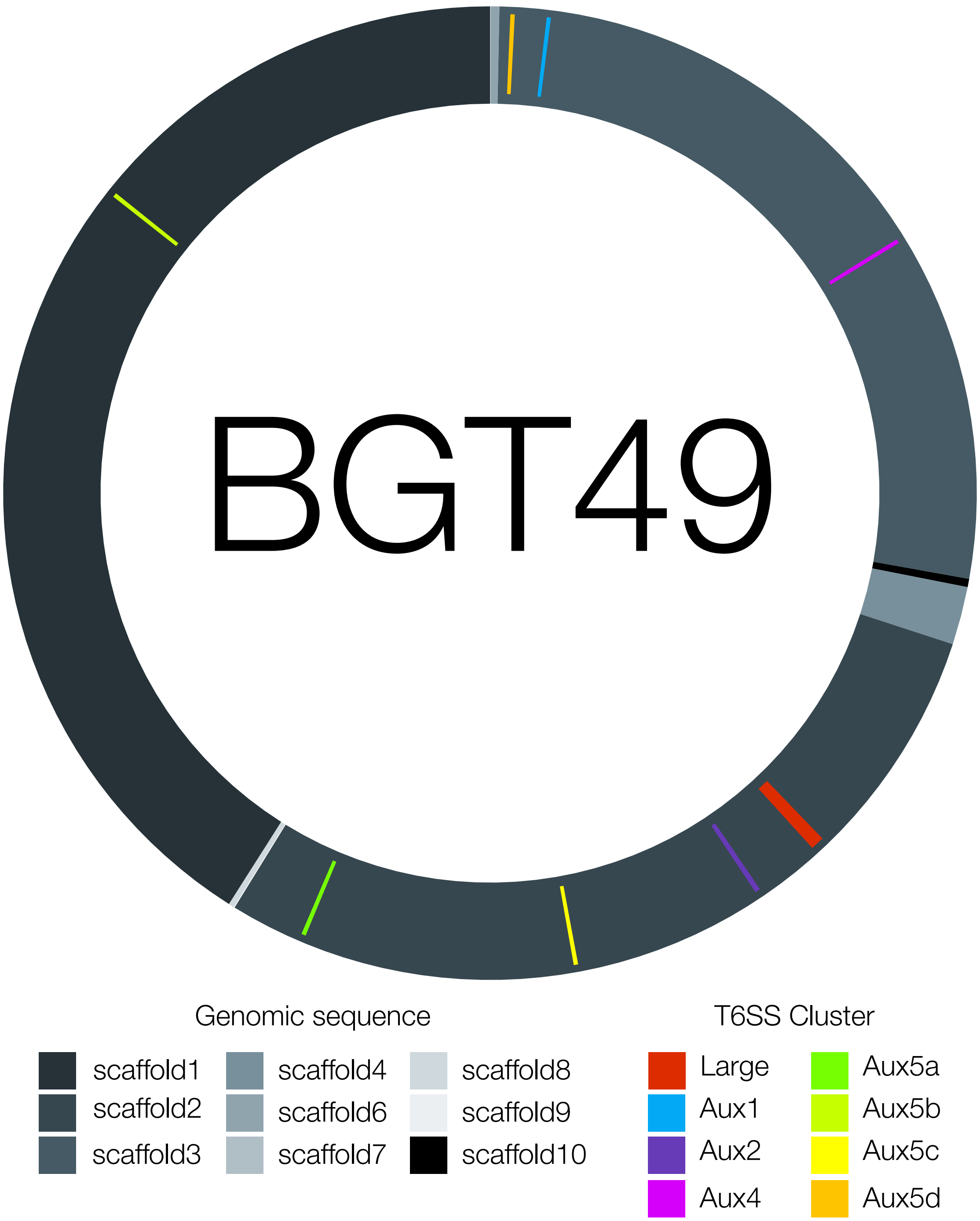

Fig. S1: Depiction of T6SS gene clusters in the BGT49 genome. In the BGT49 genome, the novel T6SS clusters Aux 4, Aux 5a, Aux 5b, Aux 5c and Aux 5d are found at distinct locations from previously known T6SS clusters.

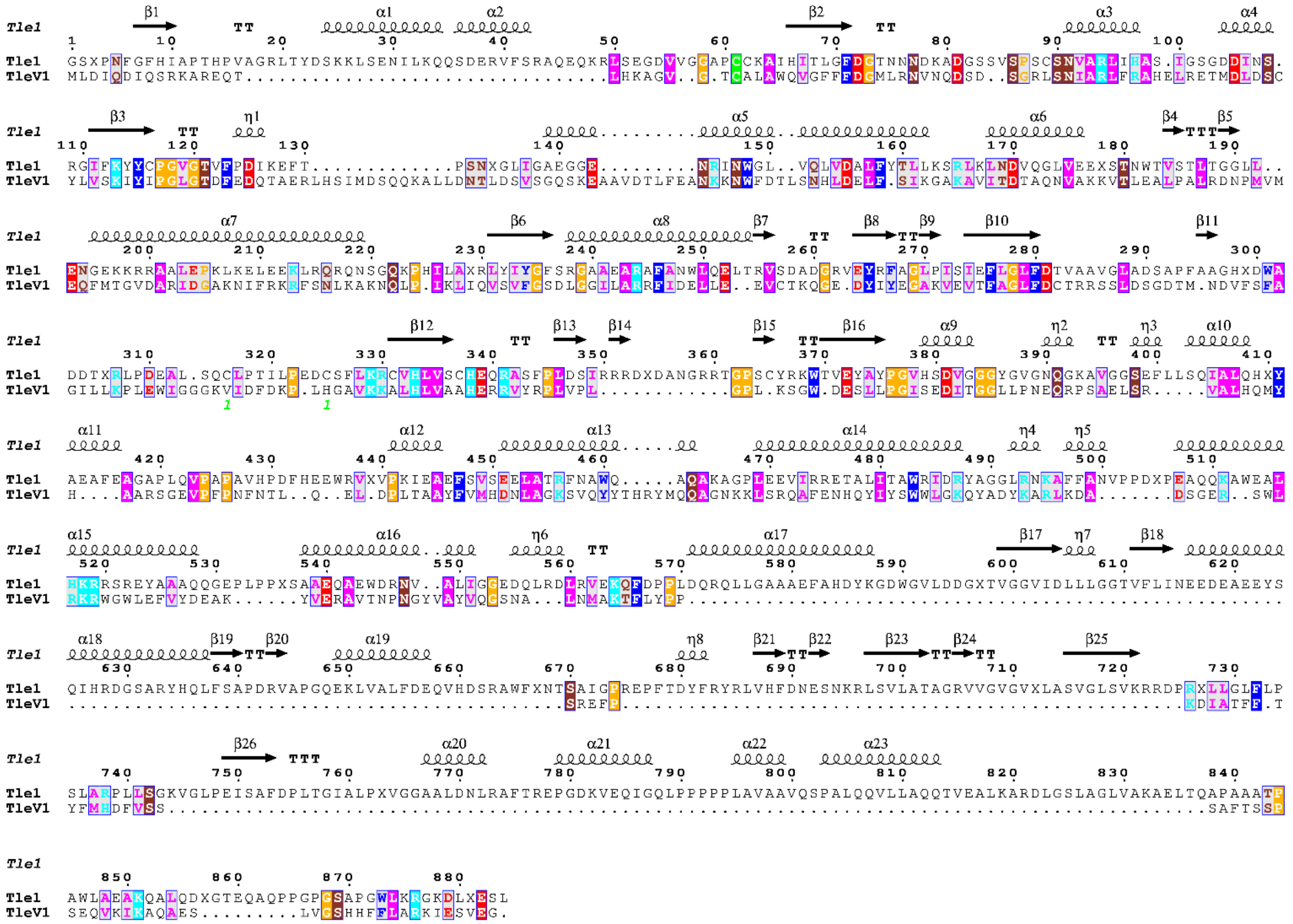

Fig. S2: TleV1 is predicted to share structural homology to the *P. aeruginosa* Tle1 T6SS lipase. Phyre2 modelling of TleV1 predicts its secondary structure is similar to Tle1, despite sharing only 19% amino acid sequence homology.

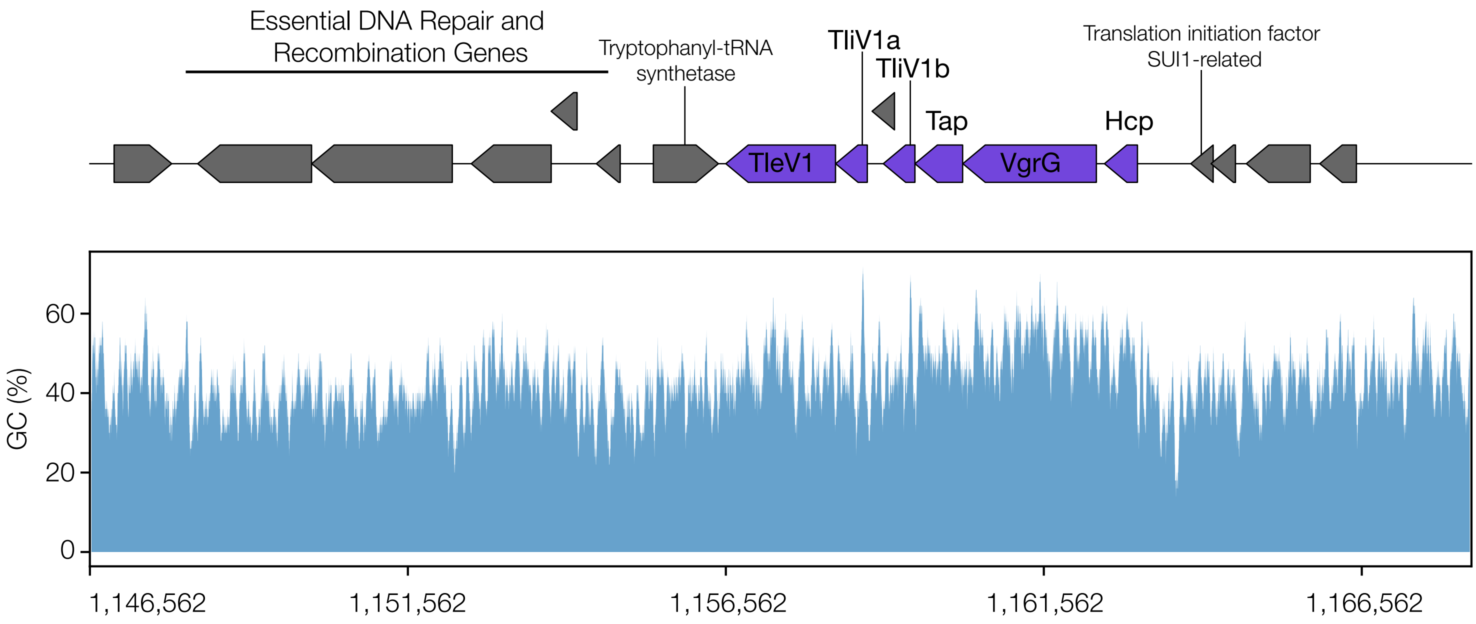

Fig. S3: BGT49 genome region containing the Aux 5a cluster. The Aux 5a cluster is flanked by genes involved in essential cell processes such as DNA repair, translation and transcription. The cluster has a similar GC content to regions flanking it.

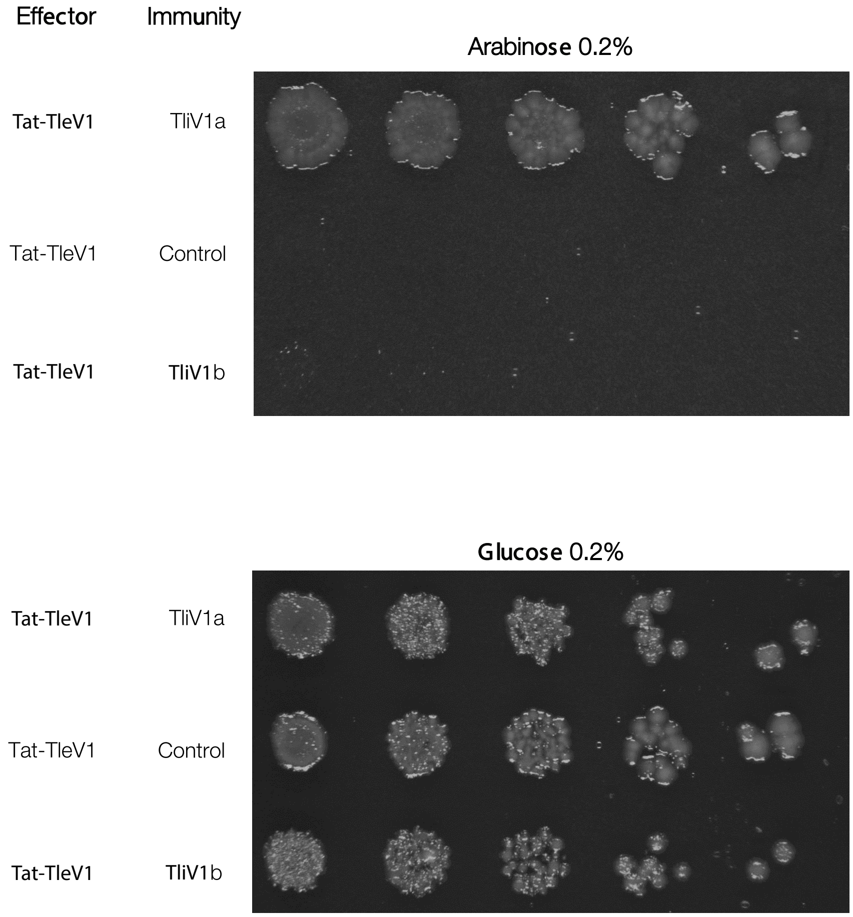

Fig. S4. Expression of the *tliV1b* gene does not neutralize the toxicity of Tat-TleV1. *E. coli* cells expressing both Tat-tleV1 and TliV1b were grown on glucose 0.2% and arabinose 0.2% (and respective antibiotics to maintain both plasmids).

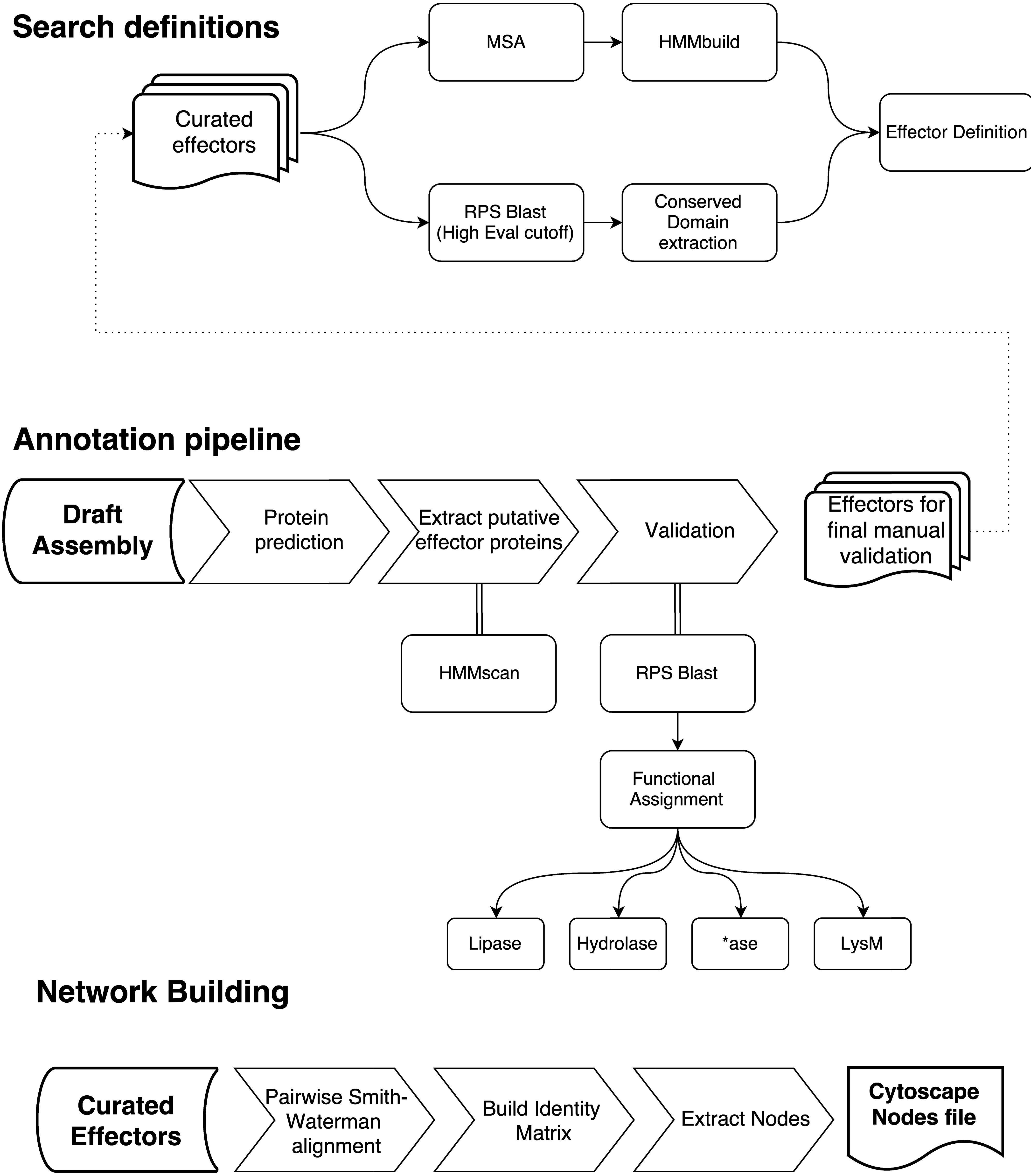

Fig. S5: Graphical description of the annotation workflow used in this study and, in part, by the T6SS predictor.
