# Supplementary material for: Analysis of Vibrio cholerae genomes identifies new type VI secretion system gene clusters
Source: Genome Biol. 2019 Aug 12;20:163. doi: 10.1186/s13059-019-1765-5 (PMC6691524; doi:10.1186/s13059-019-1765-5)
Supplement: Supplementary file 2 — Table S1. V. cholerae genomes analyzed in this study. Table S2. V. cholerae and Aeromonas strains encoding Aux 5-like clusters. Table S3. Genetically modified E. coli and V. cholerae strains and plasmids used in this study. (PDF 266 kb) [file 13059_2019_1765_MOESM2_ESM.pdf]

Table S1: *V. cholerae* genomes analyzed in this study.

Strain	Strain number	Accession	Location	Source	Year of isolation	Type VI killing activity	Coverage	Number of scaffolds	Assembled bases	N50	L50	GC %	Ns/100Kbp
**V52**	SW491	GCA_001857545.1	Sudan	Patient	1968	+	2,504x	175	3,957,367	177,630	7	47.56	13.52
**3223-74**	BGT69	GCA_001743085.1	Guam	Storm drain	1974	+	2,740x	173	4,069,095	279,340	6	47.65	5.97
**2523-87**	BGT68	GCA_001857345.1	USA (LA)	Moore swab	1974	+	1,605x	177	3,964,384	320,410	4	47.69	29.89
**3225-74**	BGT70	GCA_001857365.1	Guam	Storm drain	1974	+	2,451x	203	4,057,051	387,604	4	47.79	4.63
**3272-78**	BGT63	GCA_001857265.1	USA (MD)	Water	1977	+	2,245x	143	3,999,272	289,139	3	47.58	6.98
**2559-78**	BGT60	GCA_001857145.1	USA (LA)	Crab	1978	+	1,736x	164	4,040,166	298,103	6	47.61	17.2
**2631-78**	BGT61	GCA_001857225.1	USA (LA)	Moore swab	1978	+	1,947x	176	3,995,972	150,154	10	47.67	–
**1074-78**	BGT71	GCA_001857405.1	Brazil	Sewage	1978	+	1,644x	175	4,003,881	127,547	12	47.68	21.75
**2633-78**	BGT72	GCA_001857425.1	Brazil	Sewage	1978	+	2,714x	154	3,982,026	127,546	11	47.67	21.57
**692-79**	BGT64	GCA_001857285.1	USA (LA)	Water	1979	+	2,115x	122	3,953,095	249,947	5	47.63	14.14
**2740-80**	BGT08	GCA_001729185.1	USA (Gulf coast)	Water	1980	+	2,172x	223	4,040,634	260,656	6	47.73	14.35
**VC22**	BGT41	GCA_001729195.1	USA (FL)	Oyster	1981	+	1,650x	146	4,062,714	154,565	9	47.53	13.83
**VC48**	BGT42	GCA_001857165.1	USA (FL)	Oyster	1981	+	1,612x	176	3,954,170	114,301	11	47.57	14.57
**2512-86**	BGT62	GCA_001857245.1	USA (LA)	Moore swab	1986	+	1,380x	212	4,035,883	298,103	6	47.69	18.43
**2479-86**	BGT65	GCA_001857305.1	USA (LA)	Moore swab	1986	+	1,766x	17	4,039,476	320,139	4	47.69	4.36
**2497-86**	BGT67	GCA_001857355.1	USA (LA)	Moore swab	1987	+	2,314x	159	4,012,321	298,103	6	47.57	13.68
**857**	BGT07	GCA_001729125.1	Bangladesh	Water	1996	+	1,508x	131	4,020,975	249,952	5	47.67	15.17
**SIO**	BH2680	GCA_001857455.1	USA (CA)	Water	2000	+	1,504x	173	4,017,200	200,950	7	47.19	2.64
**TP**	BH2691	GCA_001857485.1	USA (CA)	Water	2000	+	1,551x	199	4,053,395	257,963	6	47.57	4.96
**3568-07**	EB649	GCA_001857505.1	Mexico	Queso fresco	2007	+	2,366x	172	4,095,080	106,941	16	47.37	2.34
**VC53**	BGT46	GCA_001857155.1	USA (AL)	Oyster	2009	+	2,073x	267	4,239,039	86,454	14	47.26	48.55
**VC56**	BGT49	GCA_001857175.1	USA (AL)	Oyster	2009	+	1,710x	244	4,227,097	86,314	15	47.29	14.22
**HE46**	EGT01	GCA_001857515.1	Haiti (Centre)	Gray water	2011	+	2,424x	167	4,039,421	149,375	11	47.68	35.04
**1496-86**	BGT66	GCA_001857325.1	USA (LA)	Moore swab	1986	-	1,511x	161	3,982,315	280,655	6	47.54	2.26
**C6706**	BH1514	GCA_001857435.1	Peru	Patient	1991	-	1,204x	150	4,035,736	260,568	6	47.45	36.06
**MZO-2**	BGT11	GCA_001729155.1	Bangladesh	Patient	2001	-	2,356x	161	4,001,541	255,781	5	47.51	27.96

Table S2: ***V. cholerae*** **and** ***Aeromonas*** **strains encoding Aux 5-like clusters.**

Accession	Species	Date of publication	Most similar BGT49 Aux 5 cluster	Collection metadata		
				Location	Source	Year
ATEV02000008.1	*Vibrio cholerae* VCC19	30-May-14	d	Sao Paulo, Brazil	Sewage	1994
JMBL01000033.1	*Vibrio cholerae* 1421-77 DA89	31-Jul-14	d	India	Human stool	1977
CDBJ01000027.1	*Aeromonas rivuli* MDC 2511	29-Jan-15	b	Germany	Water	2009
LT897798.1	*Vibrio cholerae* BC1071	20-Dec-17	c	Germany	Human blood	2016
PYCD01000009.1	*Vibrio cholerae* 124	18-Apr-18	d	Elista, Russia	Water	2015
QKKW01000070.1	*Vibrio sp.* 2017V-1176	10-Jul-18	d	Iowa, USA	Animal feed	2017
QEED01000041.1	*Vibrio cholerae* BD22	31-Jul-18	c	Mathbaria, Bangladesh	Water	2013
MCAZ01000167.1	*Vibrio cholerae* VN-2923	4-Sep-18	b	Germany	Water	2014
MCBA01000164.1	*Vibrio cholerae* VN-2825	4-Sep-18	b	Germany	Water	2014
MCBB01000013.1	*Vibrio cholerae* VN-2808	4-Sep-18	b	Germany	Water	2014
RHPA01000002.1	*Vibrio cholerae* O75TS	6-Nov-18	c	Missing	Missing	Missing

Table S3: Genetically modified *E. coli* and *V. cholerae* strains and plasmids used in this study.

Bacterial Strains

Strain	Description	Genotype	Reference
**NRD206**	*E. coli* MG1655	*ΔlacZY ΔaraBAD*	N. R. De Lay and J. E. Cronan. J. Biol. Chem. 282.28: 20319-28, 2007
**BH1514**	*V. cholerae* C6706	El Tor biotype O1	K. H. Thelin and R. K. Taylor, Infect. Immun. 64(7): 2853-2856, 1996
**KW25**	*V. cholerae* C6706	*ptac-qstR, ΔlacZ::Aux5a*	This study
**KW26**	*V. cholerae* C6706	*ptac-qstR, ΔvasK, ΔlacZ::Aux5a*	This study
**CC111**	*V. cholerae* C6706	*ptac-qstR, ΔlacZ::Aux5a, ΔtleV1*	This study
**JT516**	*V. cholerae* C6706	*ΔlacZ::SpecR*	This study
**CC94**	*V. cholerae* C6706	*ΔlacZ::SpecR, pSLS3*	This study
**KW18**	*V. cholerae* C6706	*ΔlacZ::SpecR, pSLS3-tliV1a*	This study
**CC95**	*V. cholerae* C6706	*ΔlacZ::SpecR, pSLS3-tat-tliV1a*	This study
**CC120**	*V. cholerae* C6706	*ΔlacZ::SpecR, pSLS3-tliV1b*	This study

Plasmids

Plasmid	Features	Reference
**pBAD18**	*pBAD* promoter, SpecR, pSLS3 origin or transfer	L.M. Guzman, et al. J. Bacteriol. 177 4121-30, 1995
**pBAD18TleV1**	*tleV1a*, SpecR	This study
**pBAD18Tat-TleV1**	*tat-tleV1a*, SpecR	This study
**pSLS3**	CmR	K. C. Tu and B. L. Bassler. Genes& dev. 21: 221-233, 2007
**pSLS3TliV1a**	*ptac-tliV1a*, CmR	This study
**pSLS3Tat-TliV1a**	*Ptac-tat-tliV1a*, CmR	This study
**pSLS3TliV1b**	*ptac-tliV1b*, CmR	This study
