# Supplementary material for: Analysis of Vibrio cholerae genomes identifies new type VI secretion system gene clusters
Source: Genome Biol. 2019 Aug 12;20:163. doi: 10.1186/s13059-019-1765-5 (PMC6691524; doi:10.1186/s13059-019-1765-5)
Supplement: Supplementary file 3 — Review history. (DOCX 31 kb) [file 13059_2019_1765_MOESM3_ESM.docx]

Review History

**First round of review**

**Reviewer 1**

**Are you able to assess all statistics in the manuscript, including the appropriateness of statistical tests used?** *There are no statistics in the manuscript.*

**Comments to author:**

The article provides a solid proof (using genetics) that the new aux5 loci indeed encodes a new T6SS dependent novel effector and immunity protein combination, which is the strength of the paper. The authors clearly show that the effector encoded at this locus can kill other cells and that one of the immunity proteins protects against this effector. Although the title of the paper suggests a focus on the discovery of new T6SS loci, this is not the case. There is a rough overview of the presence of the four auxiliary T6SS loci in a limited number of strains, without including information on their position in the genomes and how they are related to each other phylogenetically. The detection tool developed is useful, but it is not a focus of the paper and does not warrant being part of the title.

To have a more gnomic focus and not being solely a manuscript genetically describing the function of a novel effector, there needs to be a more comprehensive look at the distribution of aux4 and aux5, not just in a small set of strains. Also, there should be an investigation of the genomic locations for all T6SS clusters in different strains. Although the paper provides solid proof of the functionality of aux5, it does not really look at its diversity, or that of the other loci aux4, which is briefly mentioned but not investigated in this manuscript

Specific comments:

Why is there no mention of whether TliV1b offers protection from TleV1 ? There needs to be some information on this putative second immunity protein.

P12 l28 Is the activity of aux4 that is being investigated, but rather the activity of the effector present in the newly identified aux4 loci

Figure 2 Put the organism names at the end of the tree tips.

**Reviewer 2**

**Are you able to assess all statistics in the manuscript, including the appropriateness of statistical tests used?** *There are no statistics in the manuscript.*

**Comments to author:**

The authors have performed genomic analysis on a set of ~25 different V. cholerae genomes that their group has recently sequenced. In their analysis of T6SS-associated gene clusters, they identified a new cluster with a slightly different organization compared to other already known T6SS-associated clusters. They have confirmed that expression of this gene cluster in a heterologous Vibrio cholerae strain enables that strain to kill vibrio cholerae bacteria lacking this cluster in a T6SS-dependent manner. This is a novel finding because it is a clear demonstration of how T6SS auxiliary clusters might be acquired to expand a given T6SSs effector repertoire.

The bioinformatic analyses, however, feel like a simple "updating" of a previous study (Unterweger 2014, Nat commun, ref 32). If there's more "under the hood" in terms analytical method, it didn't really discover anything fundamentally different from this previous study (the previous study just didn't have the expanded set of genomes that are now available) - that is to say, the methods used by Unterweger et al would have also found aux 4/5 if they looked at genomes that had it. Additionally, the already existing T6SS-identifying annotation tool referenced in this study's description (SecReT6) seems to be a more comprehensive version of the T6SS Predictor developed in this study - at least it isn't restricted to Vibrio cholerae strains. Again, I can't really see "under the hood", but in terms of practical application, I don't really see the difference.

I tried going to the authors' website (vibriocholera.com) to use the program, however it did not work for me. I uploaded a sequence in the right format and clicked the button, but nothing happened (even waiting for >10 minutes)... I even tried using different browsers... all I got was a white box. Since I can't get the program to work myself, I can't really comment on its practicality/robustness/accuracy, but based the flow chart in sup fig 2, it seems to be a dressed up version of using VgrG or PAAR to blast query a genome and look at any genes nearby genes and looking for conserved enzymatic domains within these genes. This is already a common practice within the field (see this review: Lien 2017 https://doi.org/10.3389/fcimb.2017.00254) and is pretty close to what Unterweger etal did in their study. If the program is doing something more sophisticated than this, perhaps the authors could expand their explanation or give a clearer demonstration for how their program finds something other methods can't. In other words, if you are going to do the same thing, but better, you should show how it's better.

Here are some additional comments/suggestions that might be useful:

page 5, line 13, 43 - There are 48 complete, fully sequenced (closed) Vibrio cholerae strain genomes available on the NCBI database, and nearly 1000 others at the scaffold or assembly stage. It would be nice if the authors explained/rationalized why their analysis was limited to the genomes that they selected. Were they filtered for redundancy? Chosen based on genomic diversity? Geographic isolation diversity? Sequence coverage? At random? This is kind of important you're going to report data like "Feature X is found Y% of genomes."

page 7, line 25 - Can you find Aux 5 style Hcp-VgrG-TAP-effector clusters in non-vibrio species? How about this style of effector (independent of the Hcp/VgrG/TAP)? Despite the immunity proteins coming before the effectors in Aux 5 (not unheard of among T6SS effectors - e.g. tse1 from Pseudomonas aeruginosa), overall, Aux clusters 4 and 5 do have similar overall synteny as other Hcp-VgrG-TAP-effector clusters found in Vibrio and other T6SS+ organisms... Can anything be said about their evolutionary relatedness? For example, how similar are Aux 1/2 to 4 and 5? How about to other organisms... Aux 4 is described as being on a self-excisable genetic element in the study identifying it, does aux 5 have any identifiable att sites/IS elements/prophage elements around it? Can we make any guesses about what organism they came from? One of the most significant findings in this work is that the aux 5 cluster can be transferred functionally between strains, so some analysis of the sequence relatedness to other aux clusters or possible inferences about the origin of the cluster are warranted.

Figure 1a - In the figure, you show that the inner membrane complex is transferring from the inner membrane to the outer membrane. As far as we know, that's not how the T6SS works. Also, I'm not aware of any evidence that TAP chaperone proteins are multivalent with their respective effectors. In general, I don't think that this schematic diagram of the T6SS is necessary for this paper, but if it's going to be there, it should either agree with the current commonly accepted model or have clear explanations for why it is different.

Figure 1b - It would be extremely helpful if the authors included gene labels (e.g. locus tag/accession number) or at least nucleotide positions for the clusters in at least one of the genomes that they are present. Ideally, it'd be great if the authors could provide an accession number for the genes referenced.

Is there a reason why Aux clusters 3 and 5 are not shown in this figure? What about the main gene cluster which has a VgrG/effector in at least some of the T6SS genomes - is there a reason why this is not also included?

I think it would be useful to graphically show where each cluster is located within the genome (including which chromosome) to clearly show that aux 4 and 5 are distinct from aux 1/2 and from each other. Alternatively, perhaps the authors could include the genome context of each cluster (what are the neighboring genes). This is important considering several of the analyzed genomes are still just scaffolds/contigs.

Figure 2 - (just a very minor point) It would have been helpful if clusters 1 through 5 were labeled "Aux Clusters" instead of just Clusters. This would help with the differentiation from the "strains that cluster together" mentioned in the figure legend.

I think it would also be good to include the VgrG/effector from the main T6SS gene cluster, i.e. Is the evolved VgrG carrying a lysozyme domain conserved in all vibrio strains?

Figure 4b - WT C6706 is used a bit liberally here (and in the paper in general). The C6706 used here actually has a non-neutral modification (qstR*) that makes T6SS constitutively active while true wild type C6706 has an inactive T6SS in vitro. The T6SS field as a whole has tendency to do this because mutations are often needed to activate T6SS expression. This has caused a lot of unnecessary confusion/contention. Maybe use "C6706*" to indicate that the strain has been modified.

Figure 5 - Does TliV1 have a predicted secretion signal (Tat or Sec) that would already direct it to the periplasm? Also, what about tliV1b? Is this gene protective? If not, is there any explanation for this gene's function? Is it another TAP?

**Authors Response**

**Point-by-point responses to the reviewers’ comments:**

*We would like to thank the reviewers for carefully reviewing our manuscript. We are grateful for their feedback and comments and believe we thoroughly addressed all their concerns. We also used the “Track Changes” feature in the manuscript to indicate all changes we made since the first submission. Please find our responses below each lettered comment. We also included legends for the new figures.*

**Reviewer #1:**

**A.The article provides a solid proof (using genetics) that the new Aux 5 loci indeed encodes a new T6SS dependent novel effector and immunity protein combination, which is the strength of the paper. The authors clearly show that the effector encoded at this locus can kill other cells and that one of the immunity proteins protects against this effector.**

*Thank you for acknowledging our genetic experiments!*

**B.Although the title of the paper suggests a focus on the discovery of new T6SS loci, this is not the case. There is a rough overview of the presence of the four auxiliary T6SS loci in a limited number of strains, without including information on their position in the genomes and how they are related to each other phylogenetically**

*To address this comment, we have now created a circular map of the best assembly we obtained for the BGT49 genome used in this study, as shown in a new Supplementary Figure 1 and referenced on page 7, lines 154, 160-161 of the manuscript,. The map shows the relative positions of the large, Aux 1, Aux 2, Aux 4 and the four Aux 5 clusters. We also included a phylogenetic tree of the Aux 5 clusters from other bacterial species in Figure 3C (please see below as well).*

**C.The detection tool developed is useful, but it is not a focus of the paper and does not warrant being part of the title.**

*We removed the words “using a novel bioinformatic tool” and changed the wording of the title to “Analysis of Vibrio cholerae genomes identifies new, active Type VI Secretion System Clusters”.*

**D.To have a more genomic focus and not being solely a manuscript genetically describing the function of a novel effector, there needs to be a more comprehensive look at the distribution of aux4 and Aux 5, not just in a small set of strains.**

*To assess the distribution of the Aux 5 cluster, we searched across 195,536 Genbank genomes and genome sequences and created a phylogenetic tree with the obtained clusters. We found the Aux 5 cluster in 10 other V. cholerae strains (including 2 patient isolates) and an Aeromonas species. The tree comparing all four Aux 5 clusters (Aux 5a, Aux 5b, Aux 5c and Aux 5d) is now shown in Figure 3C and is discussed on page 8, lines 189-192 and page 13, lines 289-292 of the manuscript. Interestingly, Aux 5a, Aux 5b, Aux 5c and Aux 5d do not form their own a separate clade suggesting they may have been acquired as separate events by the BGT49 V. cholerae strain in this study. As in shown Supplementary Table 3 and as referenced on page 8, lines 189- 192 of the manuscript, V. cholerae strains that contain the Aux 5 cluster have been isolated from diverse geographic locations over 30 years.*

**E.Also, there should be an investigation of the genomic locations for all T6SS clusters in different strains. Although the paper provides solid proof of the functionality of Aux 5, it does not really look at its diversity, or that of the other loci aux4, which is briefly mentioned but not investigated in this manuscript.**

*We added a brief discussion about the diversity of the Aux4 cluster on pages 12-13, lines 284-285 of the manuscript. The cluster is found in hundreds of Vibrio species and therefore we believe constructing a phylogenetic tree would not be informative. So too, the following paper examined the phylogenetic diversity of a similar cluster in Vibrio strains, and is referenced (ref #33).*

*•Labbate M, Orata FD, Petty NK, Jayatilleke ND, King WL, Kirchberger PC, et al. A genomic island in Vibrio cholerae with VPI-1 site-specific recombination characteristics contains CRISPR-Cas and type VI secretion modules. Sci Rep [Internet]. 2016 Dec 15;6(1):36891.*

**F.Why is there no mention of whether TliV1b offers protection from TleV1? There needs to be some information on this putative second immunity protein.**

*TliV1a and TliV1b are 86.06% similar at the amino acid level. Both TliV1a and TliV1 have a DUF3304 domain which is present in other T6SS immunity proteins (page 14, lines 313-31). In an analogous manner to TliV1a experiments, we have now cloned the TliV1b gene and expressed it in E. coli cells that also express the arabinose-induced TleV1 effector. We also expressed TliV1b in V. cholerae target cells. Unlike TliV1a, the TliV1b gene did not rescue survival of E. coli cells expressing the TleV1 effector as shown in new Supplementary Figure 4 and as described on page 14, lines 319-321 of the manuscript. Furthermore, V. cholerae target cells carrying the TliV1b gene did not survive in killing assay competitions with C6706:Aux 5 T6SS+ cells (data not shown). As discussed on page 13, lines 302-303 of the manuscript, we observed that a short vestigial remnant that shares limited sequence homology to effectors found in Aux 5 clusters is present downstream of the TliV1b sequence and upstream of the TliV1a sequence (Figure 3a). We hypothesize that an effector might have been present at this location in the past, but that effector was replaced by the current TliV1a – TleV1 module (page 14, lines 321-325 of the manuscript). The TliV1b could have provided immunity for the effector that has been replaced but cannot provide immunity for the TleV1 effector. Maintenance of the TliV1b gene may provide defense against foreign bacteria that might still harbor such a putative lost effector. Alternatively, TleV1b may neutralize one of the other effectors found in Aux 5b, Aux 5c or Aux 5d.*

**G.P12 l28 Is the activity of aux4 that is being investigated, but rather the activity of the effector present in the newly identified aux4 loci**

*We clarified and rewrote the sentence in the manuscript (page 12, lines 285-287): “The activity of Aux 4 is beyond the scope of this study, but the ability of the cluster to be used in a T6SS-dependent manner and the biochemical function of the effector protein are currently being investigated.”*

**H.Figure 2 Put the organism names at the end of the tree tips.**

*The figure was modified to include organism names at the tree tips.*

**Reviewer #2:**

**A.The authors have performed genomic analysis on a set of ~25 different V. cholerae genomes that their group has recently sequenced. In their analysis of T6SS-associated gene clusters, they identified a new cluster with a slightly different organization compared to other already known T6SS-associated clusters. They have confirmed that expression of this gene cluster in a heterologous Vibrio cholerae strain enables that strain to kill vibrio cholerae bacteria lacking this cluster in a T6SS-dependent manner. This is a novel finding because it is a clear demonstration of how T6SS auxiliary clusters might be acquired to expand a given T6SSs effector repertoire.**

*Thank you, we also believe our finding that a foreign cluster can be used by other strains is very interesting!*

**B.The bioinformatic analyses, however, feel like a simple "updating" of a previous study (Unterweger 2014, Nat commun, ref 32). If there's more "under the hood" in terms analytical method, it didn't really discover anything fundamentally different from this previous study (the previous study just didn't have the expanded set of genomes that are now available) - that is to say, the methods used by Unterweger et al would have also found aux 4/5 if they looked at genomes that had it.**

*We cannot directly test the methods used by the Unterweger et al. (2014, Nat commun, ref 32) paper to detect novel T6SS clusters since the Methods sections does not provide sufficient detail to be replicated. We also explained the differences between methods employed by Unterweger et al. and methods employed by our T6SS Predictor in the Discussion section, page 11, lines 244-256.*

**C.Additionally, the already existing T6SS-identifying annotation tool referenced in this study's description (SecReT6) seems to be a more comprehensive version of the T6SS Predictor developed in this study - at least it isn't restricted to Vibrio cholerae strains.**

*As mentioned in the manuscript on page 11, lines 257-260, Secret6 does not identify Aux 5 or Aux4. We confirmed SecReT6 does not identify the novel clusters. As mentioned on page 20, lines 449-451 of the manuscript, we believe our tool is well optimized to detect novel T6SS clusters in V. cholerae species. We are currently examining possibilities to expand the tool to other genomes as well.*

**D.Again, I can't really see "under the hood", but in terms of practical application, I don't really see the difference.**

*A detailed explanation of the algorithm is found in the Methods section on pages 16- 17, lines 356-387 in the “Computational characterization of T6SS”, “Hidden Markov Models for effector prediction and annotation of new T6SS loci” and “T6SS predictor” sections of the methods section. A workflow schematic is also depicted in a new Supplementary Figure 5. We would also like to highlight that T6SS Predictor is now openly available, as a user-friendly online tool that can be used by anyone to scan genomes and predict novel effectors.*

**E.I tried going to the authors' website (vibriocholera.com) to use the program, however it did not work for me. I uploaded a sequence in the right format and clicked the button, but nothing happened (even waiting for >10 minutes)... I even tried using different browsers... all I got was a white box. Since I can't get the program to work myself, I can't really comment on its practicality/robustness/accuracy, but based the flow chart in sup fig 2, it seems to be a dressed up version of using VgrG or PAAR to blast query a genome and look at any genes nearby genes and looking for conserved enzymatic domains within these genes. This is already a common practice within the field (see this review: Lien 2017 https://doi.org/10.3389/fcimb.2017.00254) and is pretty close to what Unterweger etal did in their study. If the program is doing something more sophisticated than this, perhaps the authors could expand their explanation or give a clearer demonstration for how their program finds something other methods can't. In other words, if you are going to do the same thing, but better, you should show how it's better.**

*We apologize for the website issue; some software libraries employed by the algorithm did not update properly and this caused the website to be temporarily unavailable. However, website functionality is now fully restored. Please see above and the manuscript for detailed descriptions of the methods employed by the algorithm.*

**Here are some additional comments/suggestions that might be useful:**

**F.page 5, line 13, 43 - There are 48 complete, fully sequenced (closed) Vibrio cholerae strain genomes available on the NCBI database, and nearly 1000 others at the scaffold or assembly stage. It would be nice if the authors explained/rationalized why their analysis was limited to the genomes that they selected. Were they filtered for redundancy? Chosen based on genomic diversity? Geographic isolation diversity? Sequence coverage? At random? This is kind of important you're going to report data like "Feature X is found Y% of genomes."**

*We performed the genomic analysis in this study on strains that were also available to us for genetic manipulation since we used those strains for both bioinformatic and genetic studies. As shown in Supplementary Table 1, strains were obtained from a wide variety of geographic locations and sources over several decades. Although we do not make the claim that our samples are representative of the wide diversity of V. cholerae strains in the wild, we believe our collection is diverse and has uncovered novel features that are absent from well-studied V. cholerae strains.*

**G.page 7, line 25 - Can you find Aux 5 style Hcp-VgrG-TAP-effector clusters in nonvibrio species?**

*As described above in section D, to assess the distribution of the Aux 5 cluster, we searched across 195,536 Genbank genomes and genome sequences and created a phylogenetic tree with all identified cluster homologues. We found the cluster in other V. cholerae strains (including patient isolates) and an Aeromonas species. The tree comparing all four Aux 5 clusters (Aux 5a, Aux 5b, Aux 5c and Aux 5d) is now shown in Figure 3C and discussed on page 8, lines 189-194 in the manuscript.*

**H.How about this style of effector (independent of the Hcp/VgrG/TAP)?**

*As mentioned on page 7, lines 164-170 and page 13, lines 298-300 of the manuscript, based on protein structure, the effector encoded in the Aux 5a cluster most likely belongs to the Tle1 family of lipases as it is predicted with high confidence that TleV1 shares structural homology to Tle1 from Pseudomonas aeruginosa.*

**I.Despite the immunity proteins coming before the effectors in Aux 5 (not unheard of among T6SS effectors - e.g. tse1 from Pseudomonas aeruginosa), overall, Aux clusters 4 and 5 do have similar overall synteny as other Hcp-VgrG-TAP-effector clusters found in Vibrio and other T6SS+ organisms... Can anything be said about their evolutionary relatedness? For example, how similar are Aux 1/2 to 4 and 5? How about to other organisms...**

*As described above in section D, Aux 5a, Aux 5b, Aux 5c and Aux 5d do not form their own a separate clade suggesting they might have been acquired as separate events by the BGT49 V. cholerae strain in this study. As shown in Supplementary Table 2 and as referenced on page 8, lines 189-191 and page 13, lines 289-293 of the manuscript, V. cholerae strains that contain the Aux 5 cluster have been isolated from diverse geographic locations over 30 years.*

**J.Aux 4 is described as being on a self-excisable genetic element in the study identifying it, does aux 5 have any identifiable att sites/IS elements/prophage elements around it? Can we make any guesses about what organism they came from? One of the most significant findings in this work is that the aux 5 cluster can be transferred functionally between strains, so some analysis of the sequence relatedness to other aux clusters or possible inferences about the origin of the cluster are warranted.**

*As shown in new Supplementary Figure 3 and as referenced in the manuscript on page 8, lines 193-194, no significant differences were observed between the GC content in the vicinity of the Aux 5a cluster and the GC content within the cluster. Furthermore, inverted repeats or prophage-related genes were not found around the Aux 5a cluster. However, we cannot rule out that the Aux 5a cluster was integrated on the chromosome as a result of a recombination event involving large pieces of DNA. We believe there are not have enough sequences available to hypothesize the origins of the Aux 5 clusters.*

**K.Figure 1a - In the figure, you show that the inner membrane complex is transferring from the inner membrane to the outer membrane. As far as we know, that's not how the T6SS works. Also, I'm not aware of any evidence that TAP chaperone proteins are multivalent with their respective effectors. In general, I don't think that this schematic diagram of the T6SS is necessary for this paper, but if it's going to be there, it should either agree with the current commonly accepted model or have clear explanations for why it is different.**

*Both errors have now been fixed. We included the schematic to help introduce Genome Biology readers to the T6SS apparatus and the respective organization of large and auxiliary T6SS clusters.*

**L.Figure 1b - It would be extremely helpful if the authors included gene labels (e.g. locus tag/accession number) or at least nucleotide positions for the clusters in at least one of the genomes that they are present. Ideally, it'd be great if the authors could provide an accession number for the genes referenced.**

*We now included in Supplementary Figure 1 and referenced on page 7, line 154 of the manuscript a circular map of the BGT49 strain used in this study. The map shows the relative positions of the large, Aux 1, Aux 2, Aux 4 and the four Aux 5 clusters. Figure 3a also shows the relative start nucleotide positions of the four Aux 5 clusters in the BGT49 V. cholerae genome.*

**M.Is there a reason why Aux clusters 3 and 5 are not shown in this figure? What about the main gene cluster which has a VgrG/effector in at least some of the T6SS genomes - is there a reason why this is not also included?**

*As suggested, we have now added the Aux 3 cluster to Figure 1b. Since the organization of the four Aux 5 clusters is shown in figure 3A, we decided not to show it in Figure 1b to avoid repetition. Since the large cluster contains many structural and regulatory genes and since the focus of our manuscript is on novel auxiliary T6SS clusters, we decided not to include the large cluster in this figure. However, we included the large cluster lysozyme-like domains in our Figure 2 comparison.*

**N.I think it would be useful to graphically show where each cluster is located within the genome (including which chromosome) to clearly show that aux 4 and 5 are distinct from aux 1/2 and from each other. Alternatively, perhaps the authors could include the genome context of each cluster (what are the neighboring genes).**

*This is important considering several of the analyzed genomes are still just scaffolds/contigs.*

*Please see above about the inclusion of Supplementary Figure 1.*

**O.Figure 2 - (just a very minor point) It would have been helpful if clusters 1 through 5 were labeled "Aux Clusters" instead of just Clusters. This would help with the differentiation from the "strains that cluster together" mentioned in the figure legend. I think it would also be good to include the VgrG/effector from the main T6SS gene cluster, i.e. Is the evolved VgrG carrying a lysozyme domain conserved in all vibrio strains?**

*We now included the large cluster in Figure 2. As a result, we kept the label “Clusters” at the top of the figure but clarified the figure legend to make it clear that numbers 1, 2, 3, 4, 5 correspond to Auxiliary clusters, and L to Large cluster. We have changed the phrasing “strains that cluster together” to “strains that are grouped together”.*

**P.Figure 4b - WT C6706 is used a bit liberally here (and in the paper in general). The C6706 used here actually has a non-neutral modification (qstR*) that makes T6SS constitutively active while true wild type C6706 has an inactive T6SS in vitro. The T6SS field as a whole has tendency to do this because mutations are often needed to activate T6SS expression. This has caused a lot of unnecessary confusion/contention. Maybe use "C6706*" to indicate that the strain has been modified.**

*We replaced all instances of C6706 with C6706* in the manuscript, clarified the C6706* label in the legend and in the manuscript text on page 8, lines 178-181.*

**Q.Figure 5 - Does TliV1 have a predicted secretion signal (Tat or Sec) that would already direct it to the periplasm?**

*As described in the manuscript on page 14, lines 314-315, TliV1a and TliV1b are not predicted to have a periplasmic (Tat or Sec) sequence tag. Both sequences were analyzed with SignalP-5.0 and PSORTb v.3.0.*

**R.Also, what about tliV1b? Is this gene protective? If not, is there any explanation for this gene's function? Is it another TAP?**

*As described above in section F, the amino acid sequence of the two predicted proteins is 86.06% similar and both TliV1a and TliV1b have a DUF3304 domain found in other T6SS immunity proteins (page 14, lines 317-319). Tap proteins typically encode a DUF4123 domain, which is not found in TliV1b. In an analogous manner to TliV1a experiments, we cloned and expressed the TliV1b gene in E. coli cells that express the arabinose-inducible TleV1 effector and in V. cholerae target cells. Unlike TliV1a, the TliV1b gene was not found to rescue survival of E. coli cells expressing the TleV1 effector as shown in Supplementary Figure 4 and as described on page 14, lines 320-321 of the manuscript. Furthermore, V. cholerae target cells carrying the TliV1b gene were unable to survive in killing assay competitions with C6706 Aux 5 T6SS+ cells (data not shown).*

*As discussed on page 13, lines 302-303 of the manuscript, we observed a vestigial remnant that shares limited sequence homology to effectors found in Aux 5 clusters present downstream of the TliV1b sequence and upstream of the TliV1a sequence (Figure 3a). We hypothesize that an effector might have been present at this location in the past, but that effector was replaced by the current TliV1a – TleV1 module (page 14, lines 321-324 of the manuscript). The TliV1b provided immunity for the effector that has been replaced but cannot provide immunity for the TleV1 effector. The TliV1b gene could have been kept as exclusively as a defense mechanism against foreign bacteria that might still harbor the lost effector. Alternatively, TleV1b could neutralize one of the other effectors found in Aux 5b, Aux 5c or Aux 5d.*

*The following new figures and tables have been included and referenced in the revised manuscript:*

*•Figure 3C*

*•Supplementary Figure 1*

*•Supplementary Figure 3*

*•Supplementary Figure 4*

*•Supplementary Table 2*

**Second round of review**

**Reviewer 2**

I am still not convinced that the authors' new bioinformatic tool actually brings anything new to the table. I mean the authors can say that BLAST has problems and is unreliable, but at the end of the day, using BLAST to find VgrG proteins (like Unterweger did) is more than sufficient to identify aux 4 and 5 in these new Vibrio strains and their approach didn't seem to find anything other than aux 4 and 5.

That said, considering this tool has been deemphasized (i.e. removed from the title), this might not be a big deal.

Speaking of the title... I know I didn't say anything about it in the initial review, but "new active Type VI secretion system clusters" is a bit misleading since the new T6SS cluster not a new T6S system and we don't actually know if the new cluster is "active" in the organism it came from. The authors identified a new auxiliary cluster with a new effector that can be functionally introduced (or maybe reintroduced) into another Vibrio strain with an active T6SS.

Regarding the diversity of the genome selection, unless you can quantify how representative your sample is of Vibrio diversity (in which case you should show it), then statements like "Aux 3 is found in 75% of the clinical reference strains utilized but was only observed in 30% of the isolates sequenced in this study." are completely vacuous. I mean what are you trying to conclude here? Are you trying to imply that Aux3 might be relevant to clinical strains? Because that is pretty fallacious without knowing just how representative your selection is. Are you trying to say that clinical isolates are more closely related to each other than they are to environmental isolates? There's probably a better way of showing that than singling out a specific cluster.

The addition of the tree in Fig 3C was great. The revisions to figure 1 and 2 are great.

For supplementary Figure 1, is there any reason why scaffolds are organized into a single chromosome instead of two?

Also is there a way to combine the information from Sup table 2 into here? That is to say, in sup table 2, each of those vibrio strains only have 1 aux 5 cluster – which aux 5 is are they? Are they all the same one? Or are the aux5s that cluster together phylogenically also the ones that are located at the same place in the genome? Do they jump into random locations in genomes? This would further help address the discussion brought up with regard to figure 3c. Basically, I feel like additional analysis can be done here to try to understand where these clusters came from (or where they went) and if not that at least why these clusters can be functionally transferred between strains. "There are not enough sequences available to hypothesize the origins of the clusters" is a pretty big cop out.

The rest of my comments have been adequately addressed.

**Authors Response**

**Point-by-point responses to the reviewers’ comments:**

**Reviewer #2: I am still not convinced that the authors' new bioinformatic tool actually brings anything new to the table. I mean the authors can say that BLAST has problems and is unreliable, but at the end of the day, using BLAST to find VgrG proteins (like Unterweger did) is more than sufficient to identify aux 4 and 5 in these new Vibrio strains and their approach didn't seem to find anything other than aux 4 and 5. That said, considering this tool has been deemphasized (i.e. removed from the title), this might not be a big deal.**

**We believe that finding two novel gene clusters using the tool we developed significantly expanded the knowledge of genetic T6SS diversity in V. cholerae species since only three auxiliary clusters have been previously described in detail. We would like to reiterate that we developed and experimentally validated a tool that is freely available online, can detect clusters that other T6SS prediction tools such as SecReT6 miss, and has the potential to be adapted for detecting T6SS clusters in other species.*

**Speaking of the title... I know I didn't say anything about it in the initial review, but "new active Type VI secretion system clusters" is a bit misleading since the new T6SS cluster not a new T6S system and we don't actually know if the new cluster is "active" in the organism it came from. The authors identified a new auxiliary cluster with a new effector that can be functionally introduced (or maybe reintroduced) into another Vibrio strain with an active T6SS.**

**The word “active” has now been removed from the title by the editor. We also stress that in addition to introducing the cluster in another Vibrio strain, we experimentally showed that the effector encoded within the cluster has bacteriolytic activity towards V. cholerae strains in a T6SS-dependent manner.*

**Regarding the diversity of the genome selection, unless you can quantify how representative your sample is of Vibrio diversity (in which case you should show it), then statements like "Aux 3 is found in 75% of the clinical reference strains utilized but was only observed in 30% of the isolates sequenced in this study." are completely vacuous. I mean what are you trying to conclude here? Are you trying to imply that Aux3 might be relevant to clinical strains? Because that is pretty fallacious without knowing just how representative your selection is. Are you trying to say that clinical isolates are more closely related to each other than they are to environmental isolates? There's probably a better way of showing that than singling out a specific cluster.**

**The sentence “Aux 3 is found in 75% of the clinical reference strains utilized but was only observed in 30% of the isolates sequenced in this study” from page 5 was initially included simply as an observation. We did not intend to suggest Aux 3 might be more relevant to clinical strains. To eliminate any ambiguity, we changed the previous sentence to only state that “we confirmed the presence of […] the Aux 3 cluster in 30% of the isolates” (now on page 5, lines 117-118).*

**The addition of the tree in Fig 3C was great. The revisions to figure 1 and 2 are great.**

**Thank you.*

**For supplementary Figure 1, is there any reason why scaffolds are organized into a single chromosome instead of two?**

**Unfortunately, even after we sequenced the BGT49 strain using Illumina, Nanopore and PacBio technologies, we were still unable to fully assemble the genome. The illustration from Additional File 1: Fig. S1 is simply a depiction of the obtained scaffolds to show the relative genomic locations of all T6SS clusters found in strain BGT49 as requested by both reviewer #1 and reviewer #2 in their initial responses. We now clarified the figure legend to clearly state it depicts the position of the novel T6SS gene clusters relative to the previously described clusters.*

**Also is there a way to combine the information from Sup table 2 into here? That is to say, in sup table 2, each of those vibrio strains only have 1 aux 5 cluster – which aux 5 is are they? Are they all the same one? Or are the aux5s that cluster together phylogenically also the ones that are located at the same place in the genome?**

**We now added a column in Additional File 2: Table S2 to show the similarity between the four Aux 5 BGT49 clusters and those found in other strains.*

**Do they jump into random locations in genomes? This would further help address the discussion brought up with regard to figure 3c. Basically, I feel like additional analysis can be done here to try to understand where these clusters came from (or where they went) and if not that at least why these clusters can be functionally transferred between strains. "There are not enough sequences available to hypothesize the origins of the clusters" is a pretty big cop out.**

**We do not have fully assembled genomes for the strains in which the Aux 5 clusters have been found. Based on Figure 3C and our results that the Aux 5a cluster can be moved to V. cholerae strain C6706 using natural transformation (pages 8-9, lines 194- 201), we speculate V. cholerae strain BGT49 acquired the four Aux 5 clusters as independent natural transformation events from different bacterial species. As described on page 8, lines 188-193, we propose the clusters could have been integrated onto the genome of BGT49 using homologous recombination events involving long fragments of DNA. We would like to reiterate that the GC content in the vicinity of the Aux 5a cluster is similar to the GC content within the cluster and that inverted repeats or prophage-related genes are not found in the vicinity of the Aux 5a cluster (Supplementary Fig. 3).*

**The rest of my comments have been adequately addressed.**

**Thank you again for your careful and insightful comments!*
